# Medical Team Evaluation: Effect on Emergency Department Waiting Time and Length of Stay

**DOI:** 10.1371/journal.pone.0154372

**Published:** 2016-04-22

**Authors:** Juliane Lauks, Blaz Mramor, Klaus Baumgartl, Heinrich Maier, Christian H. Nickel, Roland Bingisser

**Affiliations:** 1 Department of Information and Communications Technology, University of Basel Hospital, Basel, Switzerland; 2 Freiburg Institute of Advanced Studies, University of Freiburg, Freiburg, Germany; 3 Emergency Department, University of Basel Hospital, Basel, Switzerland; Erasmus Medical Centre, NETHERLANDS

## Abstract

Emergency Departments (ED) are trying to alleviate crowding using various interventions. We assessed the effect of an alternative model of care, the Medical Team Evaluation (MTE) concept, encompassing team triage, quick registration, redesign of triage rooms and electronic medical records (EMR) on door-to-doctor (waiting) time and ED length of stay (LOS). We conducted an observational, before-and-after study at an urban academic tertiary care centre. On July 17^th^ 2014, MTE was initiated from 9:00 a.m. to 10 p.m., 7 days a week. A registered triage nurse was teamed with an additional senior ED physician. Data of the 5-month pre-MTE and the 5-month MTE period were analysed. A matched comparison of waiting times and ED LOS of discharged and admitted patients pertaining to various Emergency Severity Index (ESI) triage categories was performed based on propensity scores. With MTE, the median waiting times improved from 41.2 (24.8–66.6) to 10.2 (5.7–18.1) minutes (min; *P* < 0.01). Though being beneficial for all strata, the improvement was somewhat greater for discharged, than for admitted patients. With a reduction from 54.3 (34.2–84.7) to 10.5 (5.9–18.4) min (*P* < 0.01), in terms of waiting times, MTE was most advantageous for ESI4 patients. The overall median ED LOS increased for about 15 min (*P* < 0.01), increasing from 3.4 (2.1–5.3) to 3.7 (2.3–5.6) hours. A significant increase was observed for all the strata, except for ESI5 patients. Their median ED LOS dropped by 73% from 1.2 (0.8–1.8) to 0.3 (0.2–0.5) hours (*P* < 0.01). In the same period the total orders for diagnostic radiology increased by 1,178 (11%) from 10,924 to 12,102 orders, with more imaging tests being ordered for ESI 2, 3 and 4 patients. Despite improved waiting times a decrease of ED LOS was only seen in ESI level 5 patients, whereas in all the other strata ED LOS increased. We speculate that this was brought about by the tendency of triage physicians to order more diagnostic radiology, anticipating that it may be better for the downstream physician to have more information rather than less.

## Introduction

Increasing emergency department (ED) volume and concomitantly ED crowding represents a major problem for health care systems worldwide [1, 2]. The situation when demand for emergency services outstrips available resources can be caused by multiple factors [3]. These can generally be considered to be a combination of input, throughput and output components of ED crowding [4], which relate to individual patient and disease characteristics as well as system level factors (e.g. ED staffing, ED arrivals, and hospital census) [5]. The most relevant output component is the exit block, i.e. the patient’s inability to gain access to appropriate hospital beds in a timely manner, even though the initial investigation and treatment in the ED have been completed, and a management plan for care and the decision to admit have been made [6]. Exit block, also referred to as access block in the USA [7], can be addressed by various output interventions (e.g. bed managers, full-capacity protocols). However, as these usually operationally exceed the ED control and therefore require system-wide interventions, EDs often focus on another key area of delay: ED throughput, i.e. the period from patient arrival at the ED to hospital admission or discharge [8, 9]. Throughput interventions are meant to improve patient flow either by optimizing “front-end” operations, i.e. registration, triage, room placement and initial provider evaluation (for review see [10]), or by enhancing diagnostic testing and ED treatment [4]–processes that usually constitute the majority of a patient’s total length of stay (LOS) in the ED. Excess ED LOS highly correlates with longer hospital LOS [7], leading not only to increased health care costs [11, 12], but also to decreased inpatient satisfaction with the entire hospitalization [13]. More importantly, ED crowding is associated with poorer quality of care and increased mortality of admitted, as well as discharged patients [14–17]. It was shown [18] that more than half of the total patient turnaround time in the ED was comprised of waiting time: time away for imaging, waiting time for laboratory results, and waiting time for the first physician’s examination. We focused on this initial waiting time, as it has been described to be strongly associated with patient satisfaction [19, 20].

Ensuring that the emergency physician sees a patient early is often used as a strategy to alleviate ED crowding by improving throughput (for review see [8, 10, 21]). Various studies addressed adding a physician at triage—e.g. in the concept of the “triage liaison physician” (TLP)—to perform brief assessments and initiate diagnostic and treatment tasks [22–35]. Only few publications stratified the outcomes by patient disposition (admitted vs. discharged) or by triage levels [30, 31, 33, 34, 36]. However, it is important to know, which patient groups benefit from interventions. Therefore, we analysed LOS and initial waiting times according to triage and disposition levels.

The primary objective of this study was to assess the impact of implementing medical team evaluation (MTE) in our ED. MTE is a concept to improve front-end operations by means of team triage, assisted by quick registration, redesign of electronic medical records (EMR), and specifically designed triage rooms. The primary outcomes were initial waiting time (door-to-doctor) and ED LOS, stratified by Emergency Severity Index (ESI) triage levels and disposition (admission vs. discharge).

## Materials and Methods

### Study Design and Setting

We performed a single-centre pre-post interventional study, approved by the ethical committee of central and north-western Switzerland (EKNZ-2015-194), to assess the impact of implementing MTE in the emergency department of the University Hospital Basel, an urban 700-bed tertiary care centre in the north-western region of Switzerland. Our ED treats all patients except gynaecology and obstetrics, and ophthalmology. Children up to 16 years of age are treated in case of major trauma. In 2014, ED census was almost 48,000 cases (>30% patients admitted).

The ED operates 24 hours with eight shifts of ED staff physicians and 14 shifts of residents for 24 beds. During peak hours, hallways are used for the care of up to 10 additional patients, and the 15-bed observation unit can also be used for work-ups. A FastTrack was installed in January 2013 treating patients with minor injuries within the ED.

### ED process prior to implementation of the MTE concept

Before MTE was introduced, patients underwent a full formal registration by a registration clerk upon arrival in the ED. Patients then queued for assessment by the triage nurse, who recorded the visit type (illness vs. accident), chief complaint, the ESI level, and obtained vital signs, if necessary. After this initial triage process, patients with triage levels 3–5 were sent to the waiting room awaiting the assignment to a resident or a staff physician, who would initiate diagnostic testing and intervention as soon as a bed became available.

### Intervention: Introduction of the MTE concept

#### Team triage

Team triage was introduced together with the other parts of the MTE concept on July 17^th^ 2014: A registered triage nurse was teamed with a senior, i.e. board certified emergency physician. From the start, MTE was operated by two teams, 7 days a week from 9:00 a.m. to 10 p.m., whereby the first shift was from 9 a.m. to 6 p.m. and the second shift from 12 p.m. to 10 p.m. For triage, the German version of the ESI algorithm, validated by our group, was used [37–39]. The ESI level was assigned by triage nurses following the four decision points A to D of the ESI triage algorithm. ESI level 1 (need of an immediate life-saving intervention, decision point A), ESI level 2 (patients who should not wait due to high-risk situation, new onset of confusion, lethargy, disorientation, or severe pain or distress (decision point B)), ESI levels 3, 4, 5 (more than one, one, no resource, decision point C). Before assigning ESI level 3, vital signs must be measured (decision point D). If outside of defined limits (heart rate >100/min, respiratory rate >20/min, or oxygen saturation <92 percent), assigning ESI level 2 must be considered.

The physician’s role was to perform screening examinations, initiate diagnostic testing (e.g. ordering laboratory and radiologic examinations, requesting consultations with a specialist), and initial treatment. Additionally, non-urgent patients (typically ESI 5) were to be discharged using a “see-and-treat” approach. Patients with ESI triage levels 1 to 4 were handed over to the downstream ED physician. ESI level 1 and 2 patients were directly transferred to treatment rooms, and ESI level 3 and 4 patients had to undergo in-depth registration by a clerk and imaging, if ordered by the triage physician.

#### Designated team triage rooms

In order to facilitate rapid assessment and treatment at the point of entry, four designated team triage rooms were built next to the door, outside of the main ED treatment area–two for walk-in patients and two for patients referred by the Emergency Medical System (EMS), all facing the quick registration counter.

#### Quick registration

The prerequisite for an immediate start of treatment is a rapid registration to the ED electronic medical record (EMR) at presentation. We therefore replaced the traditional in-depth registration by a clerk with a quick initial registration by a nurse, taking down the patient’s name, gender and date of birth. If unknown, the EMR generates automatic medical record numbers allowing an immediate medical encounter with adequate electronic documentation. The so-called “quick look nurse” is also responsible for identifying patients in need of life-saving interventions and at risk (ESI 1 and 2). Bypassing team triage was especially encouraged when both teams were busy.

#### EMR redesign

The designated team triage rooms were each equipped with two computers and a printer, so that both, nurse and doctor, could individually work on the EMR, focusing on the most important information, such as history, vital signs, and initial diagnosis. Furthermore, disposition to the next caregiver had to be determined (e.g. fast track, direct boarding, high acuity bays). In order to allow simultaneous documentation by nurses and physicians, the form that was already in use prior to the introduction of MTE, was restructured. The new electronic form was introduced on June 25^th^, 2014, giving the staff some time to get used to it, before MTE was being implemented. All presenting patients were to be assessed using this new form in the EMR. Nevertheless, in some cases (e.g. ESI 1 transferred directly to resuscitation bays) information was directly entered to the EMR in order to save time.

### Selection of Participants

Initially, records of all patients presenting to the ED in 2014 were considered for inclusion except for the following: (1) records eliminated due to mistakes in registration (e.g. “double registrations”), or records of patients who left without being seen (LWBS) (2) records of patient not assessed in the ED due to early referral to another treatment site in, or affiliated with the hospital (e.g. low acuity patients seen at a family medicine centre in our hospital); (3) records of patients returning to the ED within 24h.

**Fig 1:**
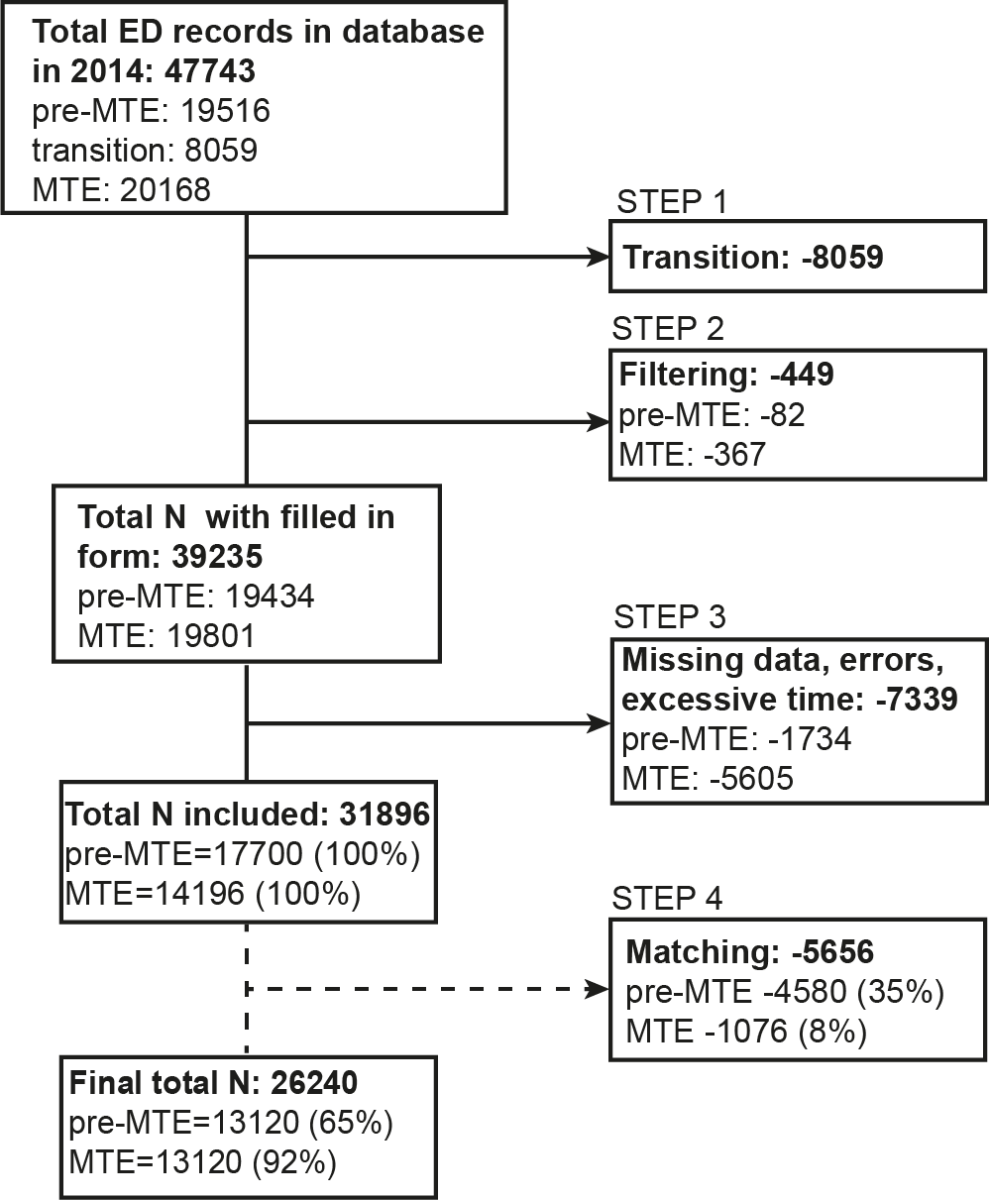
Selection of patient visits for inclusion in the study.

Then a four-step exclusion process was applied (Fig 1). In the first step, records of the transition period (June 1^st^ to July 31^st^) were eliminated. In the second step, records were filtered based on the presence of a diagnostic code in the EMR (mandatory for insurance reimbursement). In the third step, records with missing timestamps, frank errors (e.g. incorrect entries, treatment times in excess of 24 hours, or records with waiting times over 10 hours, and/or LOS over 100 hours) were eliminated. The fourth step involved removing visits that were not used in the matching process, as described under “propensity score” below.

### Methods of Measurement, Data Collection and Processing

Data collected from the ED’s EMR (ISMed NoFaSy® ProtecData) included age, gender and automatically time-stamped patient sign-in, first doctor contact, and end-of-treatment time points. The data was stored in an Oracle database v. 11.2.0.3 (Oracle Corporation, Redwood Shores, CA). Based on these parameters, the waiting time and ED LOS were calculated. The waiting time was defined as door-to-doctor time, i.e. the time from patient sign-in to first contact with a physician. To be more precise, in order for the “doctor” timestamp to be initiated, the physician had to actively select (and save) his or her name in the patient’s EMR. ED LOS was defined as door-to-end-of-treatment time, i.e. the time from patient sign-in to the time point when the patient is ready to leave the ED either for discharge (outpatients) or admission (inpatients). In addition, the patient’s ESI level and final ED disposition were recorded, allowing for further stratification of the results. Data was analysed for 5 months prior to initiation (pre-MTE period: January–May, 2014) and 5 months following MTE implementation (MTE period: August–December, 2014). The two intermediate months (June & July, 2014) of the transition period, in which a new version of the electronic patient documentation form was introduced, but the processes were still original, were excluded from the analysis. As the data were analysed anonymously, no consent was obtained from the participants. The data for the diagnostic radiology was obtained from a separate Oracle database v. 11.2.0.3. For data management the statistical software package R v. 3.1.2 (The R Foundation for Statistical Computing, Vienna, Austria) was used.

### Outcome Measures and Primary Data Analysis

Continuous data are represented as medians with interquartile ranges (IQRs). Categorical data are presented as frequency counts and percentages. To compare patient demographics between the pre-MTE and MTE periods either the chi-square test to compare differences in proportions by gender, arrival time, disposition and ESI category; or the non-parametric Wilcoxon rank-sum test to compare differences in age was used. For the comparisons of both outcome measures, i.e. the door-to-doctor time and ED LOS also the Wilcoxon rank-sum test was used, as both displayed a right-hand skewness. All data comparisons included the whole 24-hour period; hence also waiting times and LOS during the intervention period when MTE was not operating (afterhours) were taken into account. All hypothesis tests were two-sided, and p-values less than 0.05 were considered statistically significant. No adjustment was made to the overall significance level of 0.05 to account for multiple comparisons. Patients of the pre-MTE were matched with the ones of the MTE period by propensity score which, in observational studies in the medical field, is often used to balance the differences on observed covariates, thereby reducing biased estimates of treatment effects [40]. The propensity score was calculated with a logistic regression model, with age, gender, disposition and ESI category being the included covariates. The nearest neighbour match on propensity score was used to produce matched pairs for evaluation. All analyses were performed using the statistical software package R v. 3.1.2 (The R Foundation for Statistical Computing, Vienna, Austria).

## Results

During 2014 a total of 47,743 ED visits were registered in our patient administration database. With 19,516 cases being seen in the pre-MTE period (151 days) and 20,168 in the MTE period (153 days), there was a clear increase in the average number of visits per day, rising from 129 to 132 visits per day. After the exclusion process, we included a total of 31,896 visits, i.e. 17,700 visits of the pre-MTE period vs 14,196 visits of the MTE period in this study. The visits during the two-month transition period (June & July), where the new electronic form was already used, but the processes not adjusted, were excluded from the study (for further exclusion criteria see Fig 1).

As shown in Table 1, the pre-MTE and MTE samples differed only in one of the demographics dimensions after matching, i.e. the arrival time, with a slight decrease in the proportion of daytime visits (9 a.m.-10 p.m.) during the MTE period.

**Table 1:** Demographics of study patients before/after matching.

Factors	After exclusion step 3		After exclusion step 4	
	pre-MTE (n = 17700)	MTE(n = 14196)	pre-MTE (n = 13120)	MTE (n = 13120)
Age (yr), median (IQR)	49 (32–69)	50 (33–70)**	52 (34–71)	51 (33–71)
Male patients, n (%)	9383 (53.0)	7406 (52.2)	6887 (52.5)	6828 (52.0)
Arrival time 09:00–21.59, n (%)	13343 (75.4)	10626 (74.9)*	9972 (76.0)	9737 (74.2)**
Patient disposition: Discharged, n (%)	11923 (67.4)	9258 (65.2)**	8380 (63.9)	8446 (64.4)
Patient disposition: NAs, n (%)	5 (<0.01)	1 (<0.01)	/	/
ESI category 1, n (%)	179 (1.0)	124 (0.9)	121 (0.9)	124 (0.9)
ESI category 2, n (%)	3191 (18.0)	3214 (22.6)**	3115 (23.7)	3214 (24.5)
ESI category 3, n (%)	6533 (36.9)	5769 (40.6)**	5867 (44.7)	5768 (44.0)
ESI category 4, n (%)	6026 (34.0)	3725 (26.2)**	3728 (28.4)	3725 (28.4)
ESI category 5, n (%)	289 (1.6)	630 (4.4)**	289 (2.2)	289 (2.2)
ESI category NAs, n (%)	1482 (8.4)	734 (5.2)	/	/

* *p* value <0.05

** *p* value <0.01

**Fig 2:**
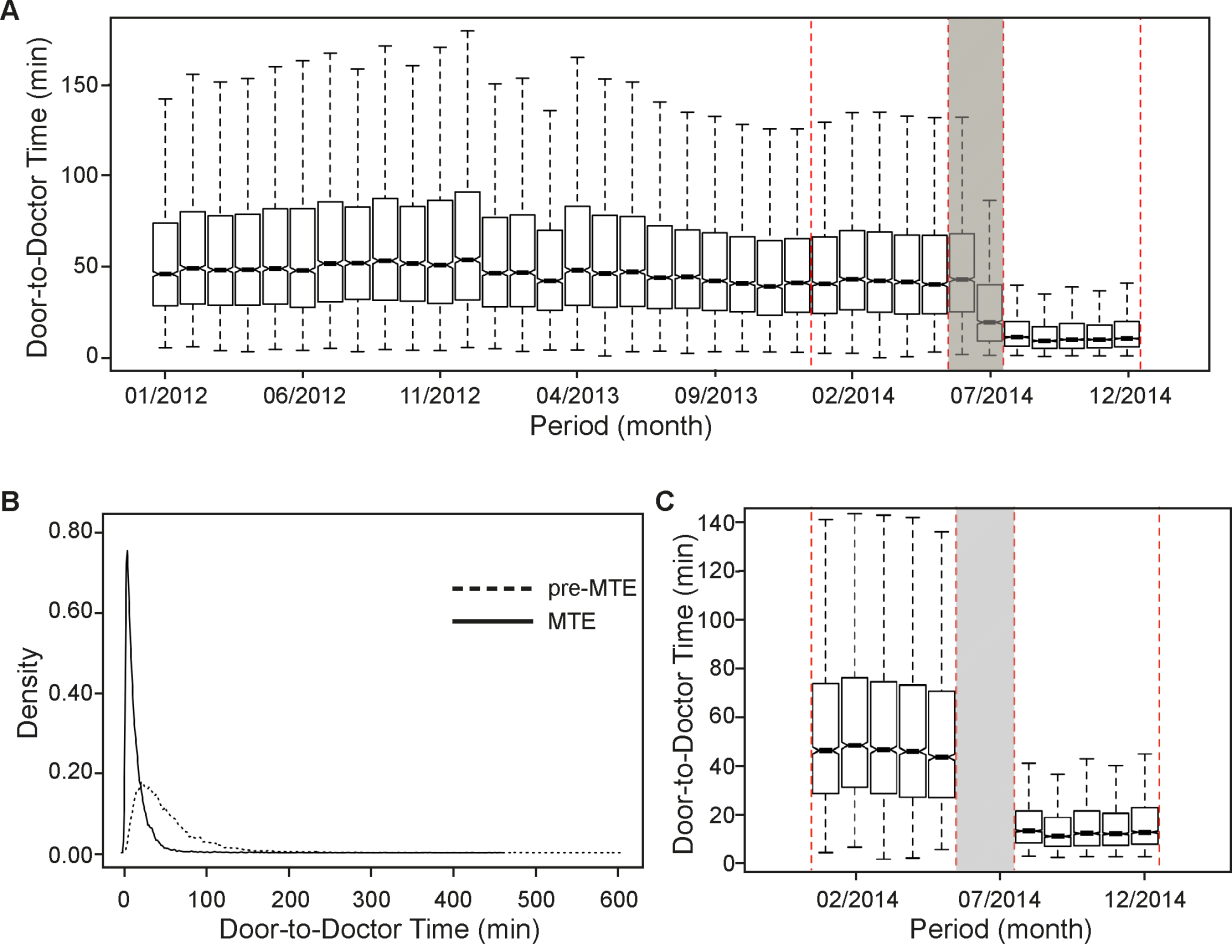
Door-to-doctor time in minutes before and after matching. (A) The box plots indicate the median, the interquartile range (box) and the smallest and largest values that are not considered outliers (whiskers, 1.5 times the interquartile range from the first and third quartile, respectively) of the data before matching. Outliers are not shown. The red-dashed-lined rectangles represent the two periods of interest, i.e. pre-MTE and MTE. (B) Density plots of matched samples. (C) Box plots of matched samples.

With the implementation of MTE, the median waiting times were significantly shorter (Fig 2), showing for the entire cohort a decrease of approximately 30 minutes (76%), (from 41 to 10 minutes; see Table 2). In contrast to the pre-MTE period in which 33% of the patients were seen by a physician within 30 minutes after presentation, within the same time frame during the MTE period, this proportion increased to 90%. Though being beneficial for all strata (when stratified by admission status or ESI category), the improvement was greater for discharged (decrease of 36 min) than admitted patients (decrease of 24 min). With a reduction from 54 to 11 min, MTE was most advantageous for ESI4 level patients. Compared to other ESI categories, upon MTE implementation, with a median of about 17 min, ESI level 5 patients had to wait longest in order to be seen by a physician.

**Table 2:** Door-to-doctor time (min) stratified by patient status and ESI category.

	Median (IQR)		
	pre-MTE (n = 13120)	MTE (n = 13120)	*p*-value
Overall	41.23 (24.78–66.62)	10.06 (5.65–18.07)	<0.01
Admitted	33.84 (19.95–55.44)	9.48 (5.18–18.12)	<0.01
Discharged	45.85 (28.08–72.93)	10.37 (5.92–18.07)	<0.01
ESI category 2	29.50 (18.38–45.88)	8.43 (4.93–15.60)	<0.01
ESI category 3	41.70 (25.23–66.10)	10.38 (5.82–18.55)	<0.01
ESI category 4	54.28 (34.23–84.70)	10.50 (5.87–18.43)	<0.01
ESI category 5	42.70 (27.85–68.80)	16.53 (11.33–23.50)	<0.01

As we could not reliably tell apart ESI level1 patients that underwent MTE from those who were immediately taken to the resuscitation area, they were excluded from the table’s individual ESI category outcomes.

**Fig 3:**
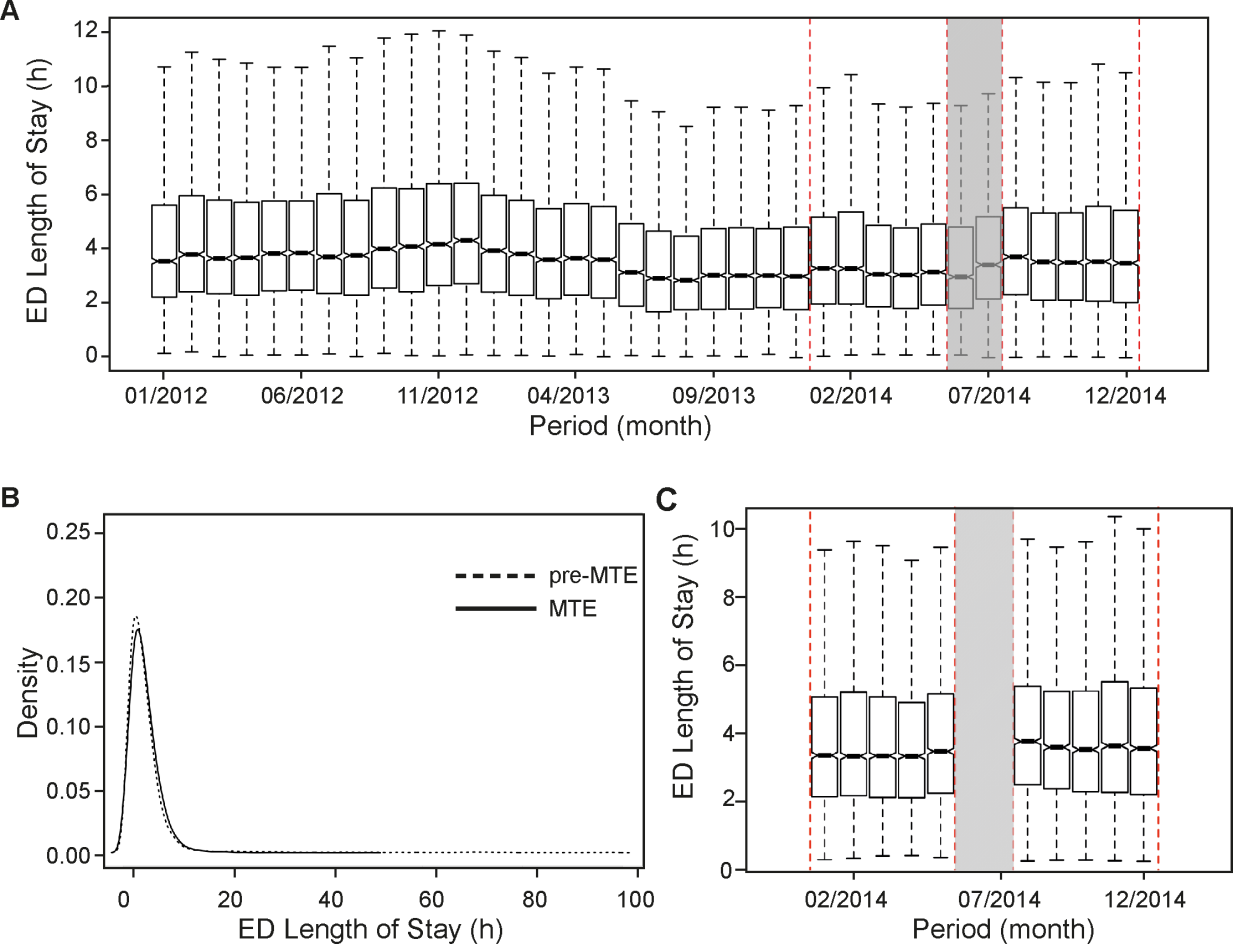
ED length of stay in hours before and after matching. (A) The box plots indicate the median, the interquartile range (box) and the smallest and largest values that are not considered outliers (whiskers, 1.5 times the interquartile range from the first and third quartile, respectively) of the data before matching. Outliers are not shown. The red-dashed-lined rectangles represent the two periods of interest, i.e. pre-MTE and MTE. (B) Density plots of matched samples. (C) Box plots of matched samples.

Upon the introduction of MTE the overall median ED LOS significantly increased (Fig 3) by 15 min (7%), rising from 3.4 to 3.7 hours (Table 3). A significant increase was observable for all strata, except for ESI5 patients, for whom the median ED LOS dropped from 1.2 to 0.3 hours, shortening their stay for 54 min (73%).

**Table 3:** ED LOS (h) stratified by patient status and ESI category.

	Median (IQR)		
	pre-MTE (n = 13120)	MTE (n = 13120)	*p*-value
Overall	3.43 (2.12–5.26)	3.68 (2.30–5.55)	<0.01
Admitted	5.01 (3.59–7.10)	5.23 (3.60–7.15)	<0.01
Discharged	2.66 (1.74–4.08)	3.00 (1.89–4.47)	<0.01
ESI category 2	4.35 (2.90–6.16)	4.57 (3.02–6.43)	<0.01
ESI category 3	4.08 (2.76–5.79)	4.32 (2.97–6.07)	<0.01
ESI category 4	2.10 (1.38–3.13)	2.40 (1.53–3.62)	<0.01
ESI category 5	1.23 (0.79–1.83)	0.33 (0.22–0.48)	<0.01

As we could not reliably tell apart ESI level1 patients that underwent MTE from those who were immediately taken to the resuscitation area, they were excluded from the table’s individual ESI category outcomes.

During the pre-MTE period a total of 10,924 orders for diagnostic radiology had been made. In the MTE period this number increased by 1,178 (11%), reaching a total of 12,102 orders (for details see S1 Table). While the number of orders for ESI1 and ESI5 patients decreased with the introduction of MTE, during the same period more imaging tests were ordered for ESI 2, 3 and 4 patients (Table 4). The biggest increase (11.7%) was seen for ESI 4 patients.

**Table 4:** Frequency of visits with at least one order for diagnostic radiology stratified by ESI category.

	pre-MTE (13120)	MTE (13120)
ESI category 1, n (%)	105 (86.8)	97 (78.2)
ESI category 2, n (%)	1969 (63.2)	2083 (64.8)
ESI category 3, n (%)	3266 (55.7)	3492 (60.5)
ESI category 4, n (%)	1304 (35.0)	1741 (46.7)
ESI category 5, n (%)	12 (4.2)	5 (1.7)

## Limitations

Several limitations need to be taken into account. The study was performed at a single academic institution. Therefore, our findings may not readily be generalized to other settings. Second, the before-after design is inferior to a prospective, randomized, controlled trial. As we introduced multiple changes simultaneously (team triage, quick registration, new triage rooms, and EMR redesign) it is difficult to discern whether a single intervention for optimizing front-end operations or a combination of them led to the improved waiting times. However, placing a physician next to the ED door is most certainly the main positive effect in terms of waiting times. Adding senior physicians would be expected to give some improvement in the parameters measured independent of MTE implementation. However, when comparing the effect of a senior work-up assessment and treatment team with the effect of an extra emergency physician without model of care, Davis [41] showed that the team-based intervention was superior to the non-team-based model.

A possible bias could have been introduced by a shift in ESI categories during the study. First, there is an ongoing trend towards higher ESI categories over the last few years in our institution. Second, training before introducing MTE could have reduced undertriage. Third, the addition of a physician to triage might have further strengthened the trend towards higher ESI levels. Moreover, in patients who should not wait, we were not able to discern the ones that underwent team triage from the ones that the “quick look” nurse had directed immediately to the main ED treatment area.

After several exclusion steps, there was a disproportionate reduction in the lower ESI levels (see S2 Table and S3 Table). This might have had an impact on overall LOS. In addition, we opted to use medians instead of average LOS times since they are less prone to be impacted by extreme values. Using averages as opposed to medians would have resulted in an overall different picture. Another notable limitation is the fact that while MTE was operating only from 9 a.m. to 10 p.m., the data collected and analysed was obtained during the complete 24 h period, which might have diluted the effect on the outcome measures. Although we tried to match the pre-MTE and MTE datasets as well as possible, a slight difference in the percentage of daytime visits still persisted, which could have had an influence on the outcome.

Additionally, we could not provide ED’s occupancy rates, as they were not yet being recorded during the time of the study. We were therefore not able to correct for occupancy-dependent variation in door-to-doctor and door-to-end of treatment times. Similarly, we were not able to extract data on LWBS or other important outcomes e.g. the number of ED return visits, patient satisfaction, time to antibiotics or time to EKG in myocardial infarction, as these are not routinely assessed. As we performed a retrospective analysis, we also did not undertake any evaluations to see whether the physicians were the rate limiting step in the LOS prior to implementing MTE.

Furthermore, our analysis was based on EMR data that may or may not accurately reflect the actual ED events [42, 43]. Therefore, we sought to validate our data by a professional external Institute (Health Care Research Institute AG, Zürich, Switzerland) [44]. As shown in S4 Table, the electronic timestamps generally overestimated door-to-doctor times. On the other hand, electronic timestamps for ED LOS reflect a more accurate picture, except for ESI4 patients (see S4 Table). It is possible that due to the familiarity with the old electronic form, the electronic timestamps of the pre-MTE period matched the observed time periods better than the ones of the MTE period, which might have had an impact on the results.

Finally, based on the data evaluated, it is not possible to discern how many patients were discharged directly from MTE or to deduct whether the observed increase in diagnostic radiology orders stemmed from the MTE or the downstream physician.

## Discussion

The main findings of our study were threefold: first, the interventions introduced in our ED lead to a reduction in waiting times (for all strata); second, a reduction was observed in ED LOS for the lowest acuity (ESI5) patients; third, there was a slight increase in ED LOS for ESI 2–4 patients.

By adding a physician to triage, waiting times i.e. door-to-doctor times are bound to improve. Except for ESI5 patients, MTE levelled out the differences in waiting times between the different strata. While in the pre-MTE period the median ESI2 and ESI4 door-to-doctor times differed by about 25 min, this difference shrank to about 2 min in the MTE period. If identified by “quick look”, the critical and emergent patients were still transferred directly into the main ED treatment area. All others were assessed by the triage team on a “first come, first serve” basis.

The reduced waiting times of ESI 2 patients are most probably due to the “quick look nurse”, who seems to fare better identifying high-risk patients at first glance than a registration clerk [45]. In general, quick look (or first impression) is a valid prognostic tool [46] promising to enhance formal triage.

A decrease in waiting time is in line with the majority of previous studies that introduced “quick” registration [47, 48] or a physician/practitioner in triage, either as an isolated intervention [25, 27, 28, 30, 31, 49], or as a combination of both strategies [36, 50]. Only a few of these studies stratified waiting times by triage levels [27, 28, 30, 36, 49]. Published data [27] stratifying outcomes according to the Manchester triage system (MTS) [51] found that patients in triage category 4 (semi-urgent) and 5 (non-urgent) had the most benefit by the team-triage approach, while the waiting times for categories 1–3 remained unchanged. Another publication [49] using MTS reported a decrease in waiting time for category 2–4 patients, even though only category 4 patients were seen by the triage team. Travers [28] used the 4-level Patient Activity Score (PACS), and analysed the waiting times for PACS2 (serious but not life threatening) and PACS3 patients only, both of which showed improved waiting times.

Both Levsky [30] and Murrell [36] examined the effect of rapid triage using the ESI triage scale on waiting times. They were the first to show an improvement in waiting time for ESI3 patients. However, this was the only patient category that underwent team triage (and this only in case if no ED beds were available). Murrell on the other hand [36] showed findings similar to our study, i.e. team triage had the highest impact on ESI4 patients.

Regarding ED LOS in our study, ESI category 5 patients, i.e. low acuity patients with non-emergent complaints and no anticipated resource utilization, were shown to benefit most from the introduction of the MTE concept. This is not surprising, given that in the pre-MTE period triage could take as long as the treatment itself. Using MTE, these patients were discharged directly from the designated team triage rooms, rendering the time in the waiting room (after triage) and the subsequent re-routing into the main ED treatment area obsolete. However, even though MTE was used for ESI5 patients as a “see and treat” concept, ESI5 were the smallest subgroup of patients. Their higher throughput could not make up for the prolonged treatment times of the other ESI levels that were responsible for the slight increase in ED LOS.

Lack of effect of an extra physician at triage on ED throughput has been described before, though mainly for patients who required either admission, or consultation by non ED-hospital staff prior to discharge [31, 41, 52, 53], i.e. patients whose ED LOS was dependent on systematic hospital-wide issues outside the control of the ED (e.g. exit block). However, as our ED LOS measure was based on the time-point when the senior physician approved the patient leaving the ED (either for discharge or admission), and not on the actual department check-out time, other factors appear to play a role in our study.

We believe that the observed slight increase in ED LOS was mainly due to the increased orders for diagnostic radiology. Unfortunately, we cannot discriminate the origin of the order, i.e. whether it was made by the triage- or by the downstream-physicians. A tendency for a triage doctor to order more imaging tests, knowing that it may be better to have more information rather than less for the colleague downstream, seems likely, but other interpretations are possible.

To a lesser extent additional factors like the slight increase in the average number of visits per day or the observed 22% rise in resuscitation bay use (see S1 Fig) from the pre- to the MTE period might also have contributed to the effect.

To our knowledge, so far Traub [35] was the only one to report a statistically significant increase in ED LOS upon the introduction of team triage (rapid medical assessment team (RMA)). When stratifying patient logs into various groups, he discovered that the situation where the patient was seen and dispositioned by only one provider, either only by the RMA (ED LOS decrease of 45%) or only by the downstream ED physician (ED LOS decrease of 9%), was superior to the one where the patient was first seen by the RMA and whose care was afterwards transitioned to the ED evaluation area physician. The group in which the patients were sequentially processed showed a worsening of ED LOS (ED LOS increase of 8%). The authors attributed these results to: 1) the inferiority of sequential processing; 2) to the lack of congruence between the work-up deemed necessary by the RMA physician and the second ED physician. In fact, over half of patients that were transitioned from the RMA to the downstream physician had additional testing ordered after evaluation by the second physician. Although generalization across different studies is extremely difficult, we cannot exclude that similar effects might have played a role for our own results. However, we find the scenario of the MTE physicians ordering more testing more likely, given that the MTE physicians were exclusively experienced senior, i.e. board certified clinicians and the downstream physicians were often residents, while there was no residency training program the RMA study. Previous studies [54] have shown that senior physicians (“attendings”) outperformed the younger ones e.g. with regard to prediction who is “sick” versus “not sick”. Future studies will be necessary to discern how the additional testing for diagnostic radiology is brought about and whether the additional tests are justified, i.e. yielding a better outcome.

In summary, the implementation of MTE resulted in an overall decrease in door-to-doctor time across all patient categories. A decrease in total ED LOS was only seen in ESI level 5 patients, whereas in all other strata the ED LOS did not improve.

## Supporting Information

S1 Fig Frequency of resuscitation bay incidents in 2014. (TIF)

S1 Table Frequency of visits with different number of orders for diagnostic radiology stratified by ESI category. (DOCX)

S2 Table Demographics of patients prior and during operation of MTE. (DOCX)

S3 Table Proportion (%) of excluded visits per group (after exclusion step 3) stratified by ESI category. (DOCX)

S4 Table Median door-to-doctor time and ED length of stay based on electronic and observed timestamps. (DOCX)
