# Supplementary figures and images for: Medical Team Evaluation: Effect on Emergency Department Waiting Time and Length of Stay
Source: PLoS One. 2016 Apr 22;11(4):e0154372. doi: 10.1371/journal.pone.0154372 (PMC4841508; doi:10.1371/journal.pone.0154372)

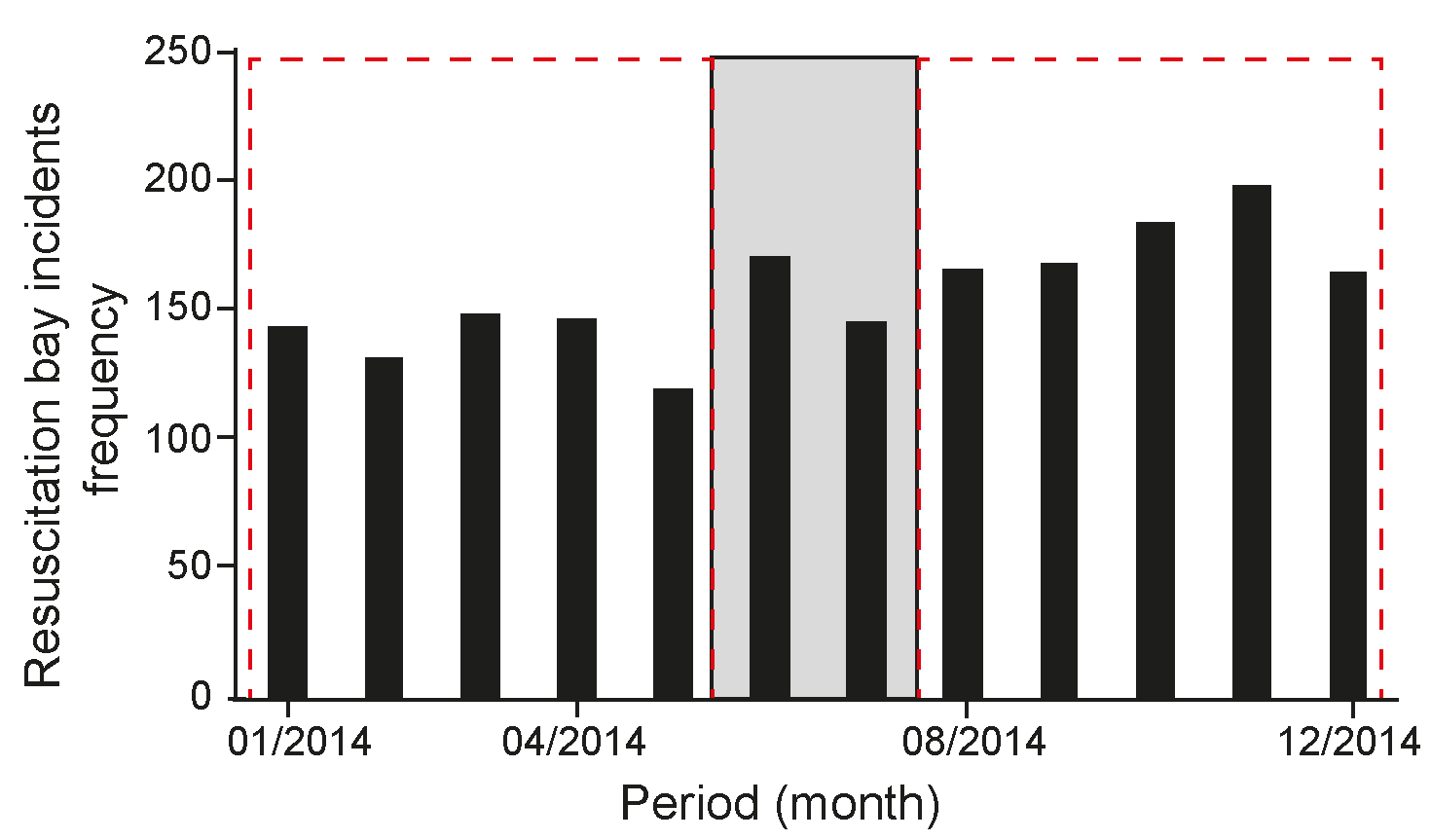

Supplement: S1 Fig — (TIF) [file pone.0154372.s001.tif]
