# Supplementary material for: Medical Team Evaluation: Effect on Emergency Department Waiting Time and Length of Stay
Source: PLoS One. 2016 Apr 22;11(4):e0154372. doi: 10.1371/journal.pone.0154372 (PMC4841508; doi:10.1371/journal.pone.0154372)
Supplement: S1 Table — (DOCX) [file pone.0154372.s002.docx]

| Pre-MTE | Number of orders for diagnostic radiology per visit | | | | | | | | | |  |
| --- | --- | --- | --- | --- | --- | --- | --- | --- | --- | --- | --- |
| ESI category | 0 | 1 | 2 | 3 | 4 | 5 | 6 | 7 | 8 | 9 | Total No of visits |
| ESI 1 | 16 | 43 | 35 | 9 | 8 | 9 | 1 | / | / | / | 121 |
| ESI 2 | 1146 | 1288 | 432 | 146 | 57 | 24 | 15 | 5 | 2 | / | 3115 |
| ESI 3 | 2601 | 1937 | 836 | 303 | 112 | 55 | 12 | 7 | 3 | 1 | 5867 |
| ESI 4 | 2424 | 619 | 525 | 108 | 41 | 11 | / | / | / | / | 3728 |
| ESI 5 | 277 | 9 | 3 | / | / | / | / | / | / | / | 289 |
|  |  |  |  |  |  |  |  |  |  |  | 13120 |
| MTE | Number of orders for diagnostic radiology per visit | | | | | | | | | |  |
| ESI category | 0 | 1 | 2 | 3 | 4 | 5 | 6 | 7 | 8 | 9 | Total No of visits |
| ESI 1 | 27 | 31 | 26 | 23 | 8 | 3 | 2 | 3 | 1 | / | 124 |
| ESI 2 | 1131 | 1303 | 510 | 157 | 65 | 32 | 11 | 2 | 3 | / | 3214 |
| ESI 3 | 2276 | 1992 | 894 | 365 | 138 | 54 | 27 | 14 | 7 | 1 | 5768 |
| ESI 4 | 1984 | 1188 | 405 | 99 | 34 | 8 | 5 | / | 2 | / | 3725 |
| ESI 5 | 284 | 5 | / | / | / | / | / | / | / | / | 289 |
|  |  |  |  |  |  |  |  |  |  |  | 13120 |
