# Supplementary material for: Medical Team Evaluation: Effect on Emergency Department Waiting Time and Length of Stay
Source: PLoS One. 2016 Apr 22;11(4):e0154372. doi: 10.1371/journal.pone.0154372 (PMC4841508; doi:10.1371/journal.pone.0154372)
Supplement: S2 Table — (DOCX) [file pone.0154372.s003.docx]

| Factors | After exclusion step 2 | | After exclusion step 3 | |
| --- | --- | --- | --- | --- |
|  | pre-MTE (n=19434) | MTE (n=19801) | pre-MTE (n=17700) | MTE (n=14196) |
| Age (yr), median (IQR) | 48 (32-69) | 48 (31-68) | 49 (32-69) | 50 (33-70) |
| Male patients, n (%) | 10326 (53.1) | 10338 (52.2) | 9383 (53.0) | 7406 (52.2) |
| Arrival time 09:00-21.59, n (%) | 14586 (75.1) | 15210 (76.8) | 13343 (75.4) | 10626 (74.9) |
| Patient disposition: Discharged, n (%) | 13468 (69.3) | 13818 (69.8) | 11923 (67.4) | 9258 (65.2) |
| Patient disposition: NAs, n (%) | 10 (<0.01) | 4 (<0.01) | 5 (<0.01) | 1 (<0.01) |
| ESI category 1, n (%) | 203 (1.0) | 242 (1.2) | 179 (1.0) | 124 (0.9) |
| ESI category 2, n (%) | 3304 (17.0) | 3926 (19.8) | 3191 (18.0) | 3214 (22.6) |
| ESI category 3, n (%) | 6930 (35.7) | 6859 (34.6) | 6533 (36.9) | 5769 (40.6) |
| ESI category 4, n (%) | 6901 (35.5) | 6893 (34.8) | 6026 (34.0) | 3725 (26.2) |
| ESI category 5, n (%) | 442 (2.3) | 780 (3.9) | 289 (1.6) | 630 (4.4) |
| ESI category NAs, n (%) | 1654 (8.5) | 1101 (5.6) | 1482 (8.4) | 734 (5.2) |

*Values do not always add up to 100% due to rounding.
