# Supplementary material for: Medical Team Evaluation: Effect on Emergency Department Waiting Time and Length of Stay
Source: PLoS One. 2016 Apr 22;11(4):e0154372. doi: 10.1371/journal.pone.0154372 (PMC4841508; doi:10.1371/journal.pone.0154372)
Supplement: S3 Table — (DOCX) [file pone.0154372.s004.docx]

| ESI Category | pre-MTE | MTE |
| --- | --- | --- |
| 1 | 0.1 | 0.6 |
| 2 | 0.6 | 3.6 |
| 3 | 2.0 | 5.5 |
| 4 | 4.5 | 16.0 |
| 5 | 0.8 | 0.8 |
| NA | 0.9 | 1.9 |

*Proportion of excluded visits (%) = (visits after exclusion step 2 – visits after exclusion step 3)/visits after exclusion step 2
