# Supplementary material for: Medical Team Evaluation: Effect on Emergency Department Waiting Time and Length of Stay
Source: PLoS One. 2016 Apr 22;11(4):e0154372. doi: 10.1371/journal.pone.0154372 (PMC4841508; doi:10.1371/journal.pone.0154372)
Supplement: S4 Table — (DOCX) [file pone.0154372.s005.docx]

|  | Door-to-doctor (hh:mm:ss) | | ED length of stay (hh:mm:ss) | |
| --- | --- | --- | --- | --- |
|  | electronic | observed | electronic | observed |
| Overall [898] | 00:16:00 | 00:09:00 | 03:50:57 | 03:44:00 |
| Admitted [262] | 00:13:53 | 00:06:00 | 05:40:02 | 05:45:30 |
| Discharged [636] | 00:16:34 | 00:10:00 | 03:17:20 | 03:10:00 |
| ESI category 1 [1] | 00:11:45 | 00:00:00 | 01:50:39 | 01:51:00 |
| ESI category 2 [177] | 00:12:46 | 00:05:00 | 04:35:28 | 04:31:00 |
| ESI category 3 [394] | 00:17:16 | 00:09:00 | 04:34:49 | 04:33:30 |
| ESI category 4 [291] | 00:16:31 | 00:11:00 | 02:44:01 | 02:21:00 |
| ESI catgeory 5 [35] | 00:25:35 | 00:17:00 | 00:29:17 | 00:26:00 |
